# Divergent host adaptation in Marburg virus: a hypothesis-generating framework for prioritizing non-traditional reservoirs

**DOI:** 10.1186/s12864-026-13256-y

**Published:** 2026-08-06

**Authors:** Qi Chen, Jingjing Hu, Siru Hu, Yuxi Wang, Weijie Chen, Shuiping Lu, Jiaxu Chen, Sijia Zhuang, Zhong Sun, Karuppiah Thilakavathy, Lufang Jiang, Chenglong Xiong

**Affiliations:** 1 Shanghai Institute of Infectious Disease and Biosecurity, Fudan University, Shanghai, 200433 China; 2 Innostellar Biotherapeutics Co. Ltd, Shanghai, 201203 China; 3 School of Public Health, Key Lab of Public Health Safety, Fudan University, Ministry of Education, Shanghai, 200433 China; 4 Shanghai Changning District Health Inspection Institute, Shanghai Changning District Center for Disease Control and Prevention, Shanghai, 200335 China; 5 School of Public Health, Kunming Medical University, Kunming, 650500 China; 6 Department of Biomedical Sciences, Faculty of Medicine and Health Sciences, Universiti Putra Malaysia, Serdang, 434000 Malaysia

**Keywords:** Marburg virus, Codon usage bias, Genomic composition, Cross-species transmission, Spillover

## Abstract

**Background:**

Although *Rousettus aegyptiacus* is the primary natural reservoir of Marburg virus (MARV), recent atypical outbreaks and viral genomic diversification suggest a highly complex host-pathogen ecology. Here, we systematically evaluated the molecular homologies between MARV and diverse mammalian taxa to identify putative reservoir hosts and guide targeted surveillance.

**Methods:**

A multi-dimensional molecular characterization of the Marburg virus (MARV) genome was performed using an integrative suite of computational approaches. Distant sequence homology was interrogated via BLAST in conjunction with a null model framework, supplemented by granular analyses of codon usage bias, nucleotide composition, and dinucleotide frequencies. For comparative genomic context, we evaluated a cohort of 63 mammalian species across the orders Rodentia, Primates, Chiroptera, and Perissodactyla.

**Results:**

Multidimensional molecular profiling showed varied genomic similarity between MARV and mammalian groups, with stable convergence observed in Perissodactyla and certain rodents across multiple codon usage indices. Notably, the effective number of codons (ENC) in *Equus caballus* (52.43) nearly matched MARV (52.44), and *Equus asinus* exhibited the lowest average RSCU Manhattan distance to the virus (0.25). Additionally, MARV’s CpG suppression ($\:{\rho\:}_{CpG}$ = 0.48) was consistent with the CpG depletion levels characteristic of the studied mammalian taxa.

**Conclusion:**

Through the lens of computational biology, this study identifies putative host cohorts within the orders Perissodactyla and Rodentia that share distinct genomic, codon-usage, and dinucleotide-frequency homologies with MARV. These findings establish high-priority targets for downstream ecological field monitoring, seroepidemiological surveys, and experimental validation, ultimately providing a scientific blueprint to refine zoonotic surveillance networks under the global ‘One Health’ paradigm.

**Supplementary Information:**

The online version contains supplementary material available at https://doi.org/10.1186/s12864-026-13256-y.

## Background

Marburg virus (MARV) is a member of the Filoviridae family and is characterized as a single-stranded negative-sense RNA virus [1]. This virus is responsible for Marburg virus disease (MVD), which clinically manifests as acute hemorrhagic fever, accompanied by symptoms such as high fever, headache, muscle pain, bleeding tendency, and multi-organ failure. The case fatality rate of MVD ranges from 20% to 90% [2, 3], representing a significant threat to public health. The MARV genome spans approximately 19 kb in length and encodes seven structural proteins, including the nucleoprotein (NP), viral polymerase (L), and glycoprotein (GP) [4]. Among these, GP plays a crucial role in facilitating viral entry into host cells by binding to cell surface receptors, notably Niemann-Pick C1 (NPC1) [5]. As a zoonotic pathogen, MARV is predominantly found across the African continent. Its high infectivity and lethality have prompted the World Health Organization (WHO) to classify it as a pathogen of priority concern, underscoring the necessity for urgent, in-depth research to bolster prevention and control measures.

The epidemiological history of the MARV dates back to 1967, when simultaneous outbreaks were reported in Marburg and Frankfurt, Germany, and Belgrade, Yugoslavia, resulting in 31 infections and 7 fatalities [6]. Subsequent outbreaks have been documented in Ghana, Kenya, South Africa, Angola, Tanzania, the Democratic Republic of the Congo, Uganda, and Rwanda [7–14]. Despite being limited in scale, the virus’s high fatality rate and potential for community transmission prompted a pre-alert response from the WHO. The concentration of these outbreaks in sub-Saharan Africa underscores the urgent need to continually strengthen surveillance and early warning systems.

Although *Rousettus aegyptiacus* is established as the principal natural host of Marburg virus (MARV) [15], current epidemiological and genetic indices point to a more intricate reservoir ecology and regional transmission paradigm. Notably, recently affected areas in West Africa, including Guinea (2021) and Ghana (2022), lie within the bioclimatic envelope of *R. aegyptiacus*; however, historical active surveillance, viral screening, and genomic characterization within local bat populations remain severely limited [6, 16, 18, 19]. Outbreaks in these novel foci may thus stem from altered local bat population dynamics, regional genetic drift, surveillance gaps, or unrecognized spillover pathways [17, 19]. Furthermore, substantial genetic heterogeneity exists between select clinical isolates—most notably the Angola isolate—and wild reservoir-derived strains, characterized by marked sequence variations in the VP35 and GP gene loci. These genomic disparities likely underscore distinct viral lineage differentiation or localized evolutionary adaptation [8]. Accordingly, a comprehensive identification of MARV-associated host groups and their regional ecological niches is essential for establishing robust sentinel-based early warning systems and mitigating the risk of zoonotic transmission.

In this study, we leveraged a multi-pronged computational genomics pipeline—encompassing sequence homology alignments validated by null models, multidimensional codon usage patterns, and dinucleotide frequency constraints—to systematically delineate the molecular relationships between MARV and the mammalian orders Chiroptera, Perissodactyla, Rodentia, and Primates. This work provides a rigorous conceptual foundation for modeling viral spillover dynamics, elucidating complex multi-host transmission networks, and informing evidence-based, source-control interventions within the global ‘One Health’ framework.

## Methods

### Data sources

The Marburg virus (MARV) complete genomes sequences information was downloaded from the Nucleotide database at NCBI, specifically for the following entries: “Marburg marburgvirus isolate Ravn virus/H.sapiens-tc/KEN/1987/Kitum Cave-810040, complete genome” (NC_024781.1) and “Marburg virus - Musoke, Kenya, 1980, complete genome” (NC_001608.3). Additionally, public databases were queried for potential natural host species, including primates, chiropterans, and rodents in Africa, to obtain their coding sequence (CDS) data wherever available.

### Homology analysis and null-model validation

The MARV reference genome (NC_024781.1) and the divergent sequences between NC_001608.3 and NC_024781.1 were divided into 300-nt fragments. Homology was assessed via BLASTn (max target: 5,000; E-value: 0.1; word size: 11), retaining alignments with Perissodactyla and Rodentia ≥ 50 nt in length. A null model was established using Monte Carlo substitution; specifically, 20 composition-matched random sequences were generated for each positive fragment and re-analyzed using BLASTn. p-values were derived using empirical formulas to test if match frequencies were significantly higher than random expectation.

### Phylogenetic analysis

Multiple sequence alignment of the target sequences was performed using MAFFT. A phylogenetic tree was subsequently inferred with IQ-TREE under the maximum-likelihood (ML) framework. Nodal support was evaluated using 1,000 ultrafast bootstrap (UFBoot) replicates and 1,000 SH-like approximate likelihood ratio tests (SH-aLRT). The resulting tree was visualised and annotated using the Interactive Tree of Life (iTOL) platform.

### Analysis of codon usage bias

Coding sequences (CDS) for 53 mammalian species native to Africa—spanning the orders Chiroptera, Primates, and Rodentia—were retrieved from the NCBI Nucleotide database. To capture broader evolutionary relationships, CDS for an additional 10 species within Rodentia and Perissodactyla were selected based on sequence homology identified via Basic Local Alignment Search Tool (BLAST) queries. Strict quality control criteria were applied to the dataset: sequences containing ambiguous nucleotides (N) or mapping to mitochondrial and ribosomal RNA genes were excluded from subsequent analyses.

Relative synonymous codon usage (RSCU) values were computed using CodonW (v1.4.2). To account for the compositional nature of codon usage data, the resulting RSCU matrix was subjected to a centered log-ratio (CLR) transformation. Dimensionality reduction was subsequently performed on the CLR-transformed matrix via Principal Component Analysis (PCA) in Python to visualize codon-usage clustering patterns across host taxa.

To quantify the discrepancy in codon usage profiles between the 63 species of mammals and Marburg virus (MARV), the average Manhattan distance was calculated in R (v4.5.0) using the following formula:

$$\:\mathrm{D=}\frac{\mathrm{1}}{\mathrm{59}}{\sum\:}_{\mathrm{i=1}}^{\mathrm{59}}\mid{\mathrm{RSCU}}_{\mathrm{i,host}}\mathrm{-}{\mathrm{RSCU}}_{\mathrm{i,MARV}}\mid$$

where * i* represents the 59 synonymous codons analyzed (excluding stop codons and single-codon amino acids, i.e., Met and Trp).

Nucleotide compositional metrics, including overall GC content, GC content at the third codon position ($\:{\mathrm{GC}}_{\mathrm{3s}}$), and the effective number of codons (ENC), were calculated using CodonW. An *ENC--GC3s* plot analysis was employed to evaluate the relative contributions of mutational pressure and natural selection in shaping codon usage bias. Additionally, the Codon Adaptation Index (CAI) was calculated via CodonW to assess the translational adaptation efficiency of MARV within different candidate genomes.

The relative abundance of dinucleotides ($\rho_{\mathrm{XY}}$) was calculated using standard formulas, with a specific focus on CpG and UpA suppression signatures. To evaluate the statistical significance of the observed CpG suppression, a null model was constructed by generating 1,000 randomized sequences per genome. These synthetic sequences maintained the identical single-nucleotide composition of the wild-type sequences. Empirical distributions derived from this null model were utilized to calculate empirical p-values for statistical testing.

All pipeline implementation, statistical computations, and data visualizations were executed using Python and R (v4.5.0). To ensure scientific transparency and reproducibility, the complete analytical code, specific parameter configurations, and exemplar datasets have been deposited in a public GitHub repository (https://github.com/qccc1119/MARV-analysis).

## Results

### BLAST-based sequence homology analysis and null-model validation

**Fig. 1:**
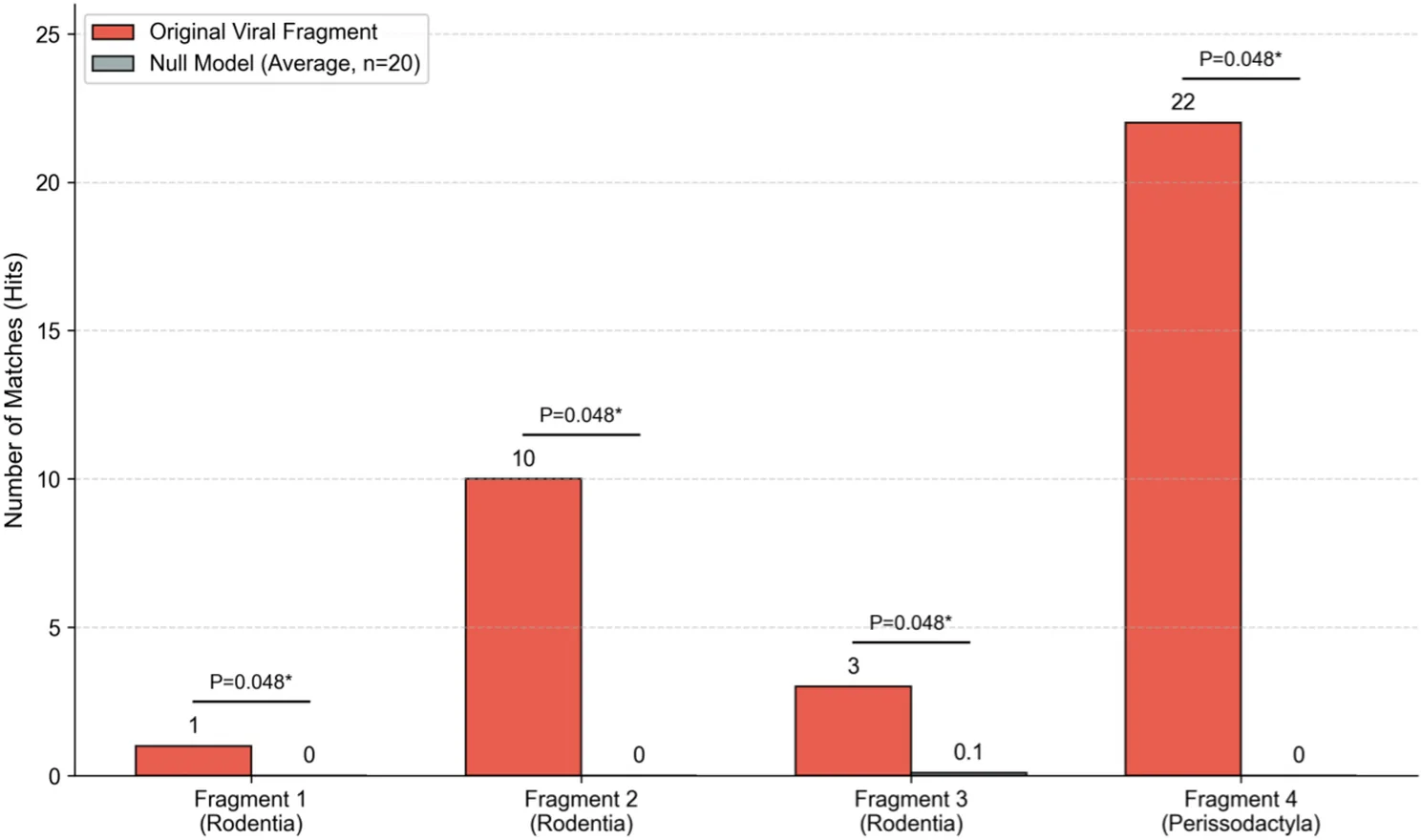
Null-model-based statistical significance of genomic alignments between MARV fragments and mammals. Red bars denote the observed number of genomic alignments for the original viral fragments within the mammalian database; grey bars represent the expected mean alignment counts based on a null model derived from nucleotide composition (n = 20 randomized sequences per fragment). Observed alignment counts for all four fragments—fragments 1–3 (targeting Rodentia) and fragment 4 (targeting Perissodactyla)—significantly exceeded stochastic expectations (p = 0.048). Analyses were conducted using the MARV reference sequence NC_024781.1

A total of 64 fragments generated from the NC_024781.1 reference sequence were subjected to BLASTn alignment against a mammalian database, identifying four valid matches: three associated with Rodentia and one with Perissodactyla. Validation via the null model demonstrated robust statistical support: fragments 1, 2, and 3 (mapping to 1–300 nt, 901–1200 nt, and 9901–10200 nt in Rodentia, respectively) yielded 1, 10, and 3 matches, while fragment 4 (mapping to 18601–18900 nt in Perissodactyla) yielded 22 matches. In all cases, the alignment frequencies significantly exceeded stochastic expectations (all *p* = 0.048) (Fig. 1 and Additional file 1: Table S1).

Phylogenetic reconstruction was performed on rodent and perissodactyl sequences (≥ 50 nt) identified through distant-homology BLAST searches. The resulting topology demonstrates that the Musoke and Ravn strains of MARV share a certain degree of sequence homology with diverse mammalian species. These include rodents (*Arvicola amphibius*, *Microtus oregoni*, *Peromyscus californicus*, and *Microtus ochrogaster*) and the perissodactyls *Equus asinus* and *Equus przewalskii* (Fig. S1 and Additional file 1: Table S2).

### Characterization of codon usage bias

**Fig. 2:**
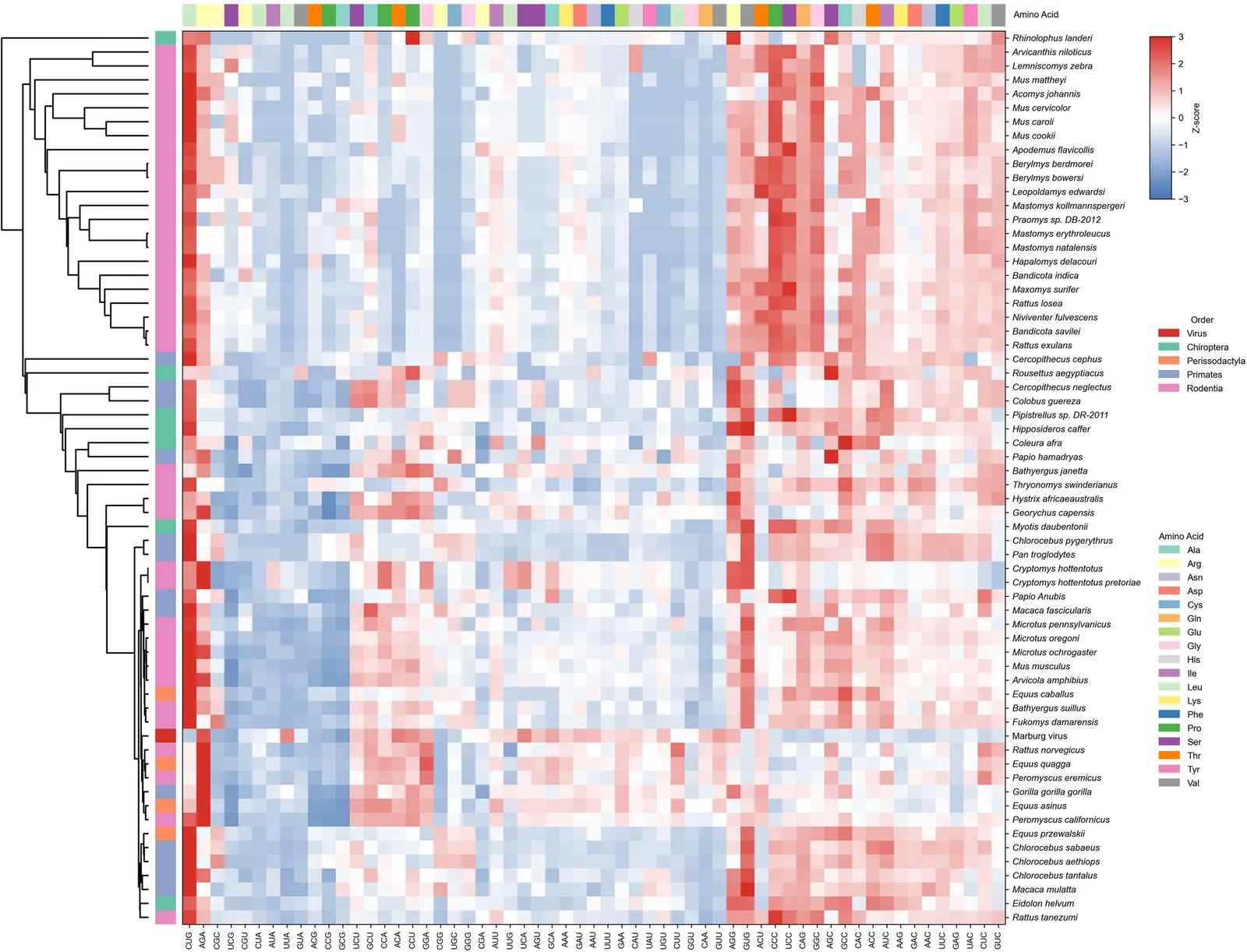
Hierarchical clustering heatmap of RSCU based on the CLR. Rows and columns represent mammalian species and synonymous codons, respectively. Cell color intensity denotes the Z-score-normalized frequency of each codon within a specific genome. Hierarchical clustering indicates that the Marburg virus (MARV) groups primarily with select rodent species, followed by perissodactyls and primates. MARV reference sequence: NC_024781.1

CLR-based hierarchical clustering reveals that MARV occupies an intermediate outgroup position between the orders Rodentia and Perissodactyla. The virus clusters most closely with *Rattus norvegicus* (Rodentia), three Equus species (*E. caballus*, *E. quagga*, and *E. asinus*), and two members of the genus Peromyscus (*P. eremicus* and *P. californicus*) (Fig. 2).

**Fig. 3:**
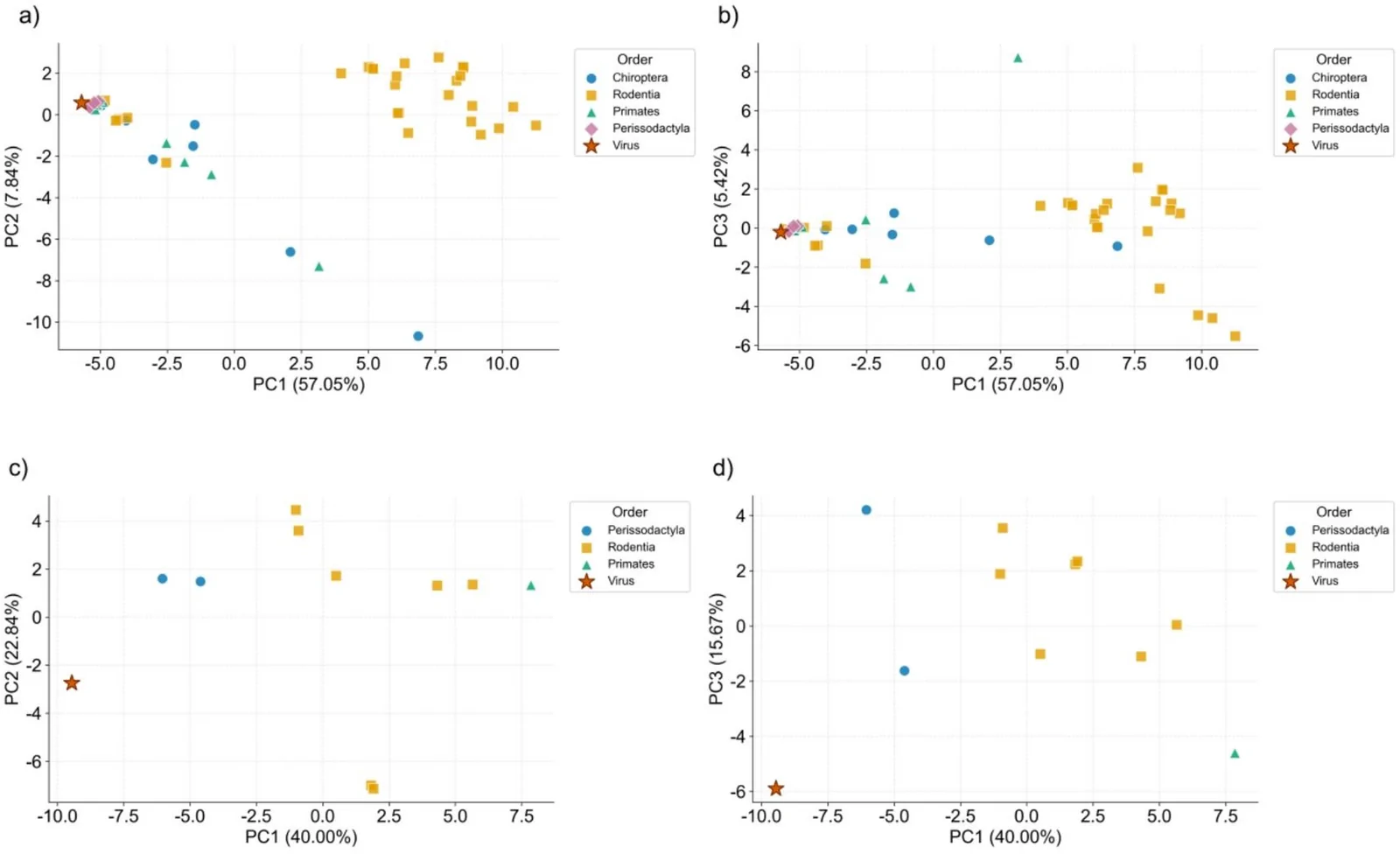
PCA of codon usage bias in MARV and mammals utilizing CLR transformation. (**a**, **b**) Global PCA projections (PC1 vs. PC2 and PC1 vs. PC3) showcasing the macroscopic distribution of MARV (red stars) relative to 63 mammals in the codon usage landscape. (**c**, **d**) Focused PCA encompassing the ten most proximal species based on minimal Euclidean distance in the 3D CLR space. Results demonstrate that MARV exhibits high genomic affinity with Perissodactyla (blue dots) and certain Rodentia (yellow squares) regarding overall codon signatures. MARV reference sequence: NC_024781.1

Global CLR-PCA analysis characterizes MARV as a significant outlier (PC1$\:\approx\:\:$-5.2), positioned beyond the negative boundary of all examined mammalian orders. Rodentia are distributed along the positive PC1 axis, whereas Chiroptera, Primates, and Perissodactyla cluster along the negative axis. Evaluation of the ten nearest-neighbor species indicates that two members of the order Perissodactyla (*Equus asinus* and *Equus quagga*) exhibit the highest proximity to MARV (Fig. 3).

**Fig. 4:**
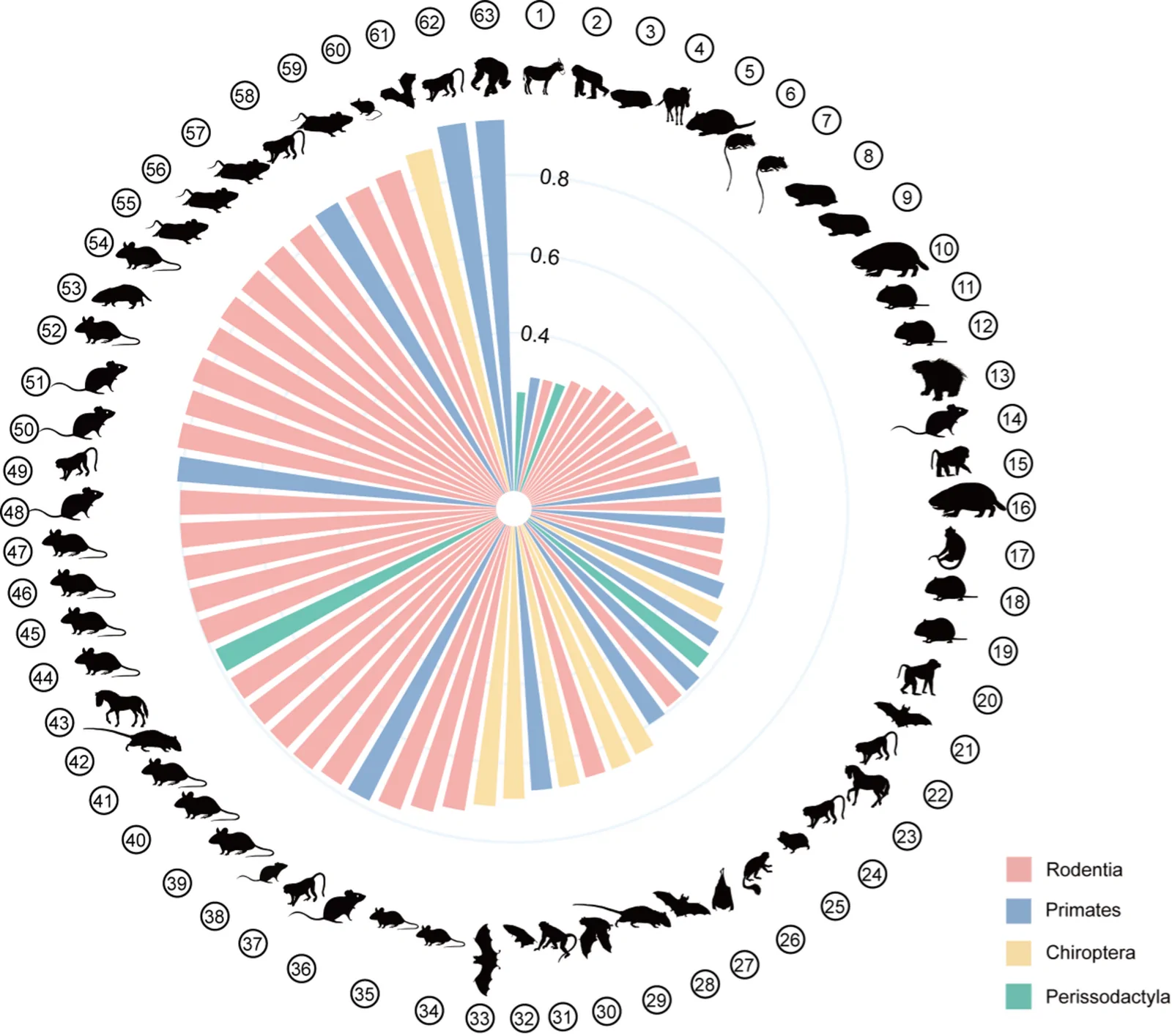
Manhattan distance analysis of codon usage preferences between MARV and various mammals The radial bar chart illustrates the average Manhattan distance in codon usage between 63 mammalian species and the MARV. The length of each bar corresponds to the average Manhattan distance between that specific species and the MARV. This figure demonstrates that certain species within the orders Rodentia (pink) and Perissodactyla (green) exhibit the closest similarity to the codon usage patterns of the MARV, while Primates (blue) and the virus’s host order, Chiroptera (yellow), display the greatest divergence. The MARV sequence analyzed is: NC_024781.1. The circled numbers in the figure correspond to the following species:1, *Equus asinus*; 2, *Gorilla gorilla gorilla*; 3, *Georychus capensis*; 4, *Equus quagga*; 5, *Rattus norvegicus*; 6, *Peromyscus californicus*; 7, *Peromyscus eremicus*; 8, *Cryptomys hottentotus*; 9, *Cryptomys hottentotus pretoriae*; 10, *Bathyergus janetta*; 11, *Arvicola amphibius*; 12, *Microtus ochrogaster*; 13, *Hystrix africaeaustralis*; 14, *Mus musculus*; 15, *Papio Anubis*; 16, *Bathyergus suillus*; 17, *Macaca fascicularis*; 18, *Microtus oregoni*; 19, *Microtus pennsylvanicus*; 20, *Papio hamadryas*; 21, *Coleura afra*; 22, *Chlorocebus tantalus* 23, *Equus caballus*; 24, *Cercopithecus neglectus*; 25, *Fukomys damarensis*; 26, *Colobus guereza*; 27, *Rhinolophus landeri*; 28, *Eidolon helvum*; 29, *Rattus tanezumi*; 30, *Myotis daubentonii*; 31, *Macaca mulatta*; 32, *Rousettus aegyptiacus*; 33, *Hipposideros caffer*; 34, *Hapalomys delacouri*; 35, *Bandicota savilei*; 36, *Mus mattheyi*; 37, *Chlorocebus aethiops*; 38, *Rattus exulans*; 39, *Arvicanthis niloticus*; 40, *Bandicota indica*; 41, *Niviventer fulvescens*; 42, *Rattus losea;* 43, *Equus przewalskii*; 44, *Apodemus flavicollis*; 45, *Berylmys berdmorei*; 46, *Berylmys bowersi*; 47, *Maxomys surifer*; 48, *Mus cervicolor*; 49, *Chlorocebus sabaeus*; 50, *Mus caroli*; 51, *Mus cookii*; 52, *Lemniscomys zebra*; 53, *Thryonomys swinderianus*; 54, *Leopoldamys edwardsi*; 55, *Mastomys erythroleucus*; 56, *Mastomys kollmannspergeri*; 57, *Mastomys natalensiv*; 58, *Cercopithecus cephus*; 59, *Praomys sp. DB − 2012*; 60, *Acomys johannis*; 61, *Pipistrellus sp. DR- 2011*; 62, *Chlorocebus pygerythrus*; 63, *Pan troglodytes*

*Equus asinus* has the lowest average Manhattan distance of 0.25. *Gorilla gorilla gorilla*(0.29), *Georychus capensis*༈0.29༉ and *Equus quagga*༈0.29༉ also exhibit relatively small RSCU-biased distances from the MARV (Fig. 4 and Additional file 1: Table S3).

### Characterization of the nucleotide composition of MARV and its mammals

**Fig. 5:**
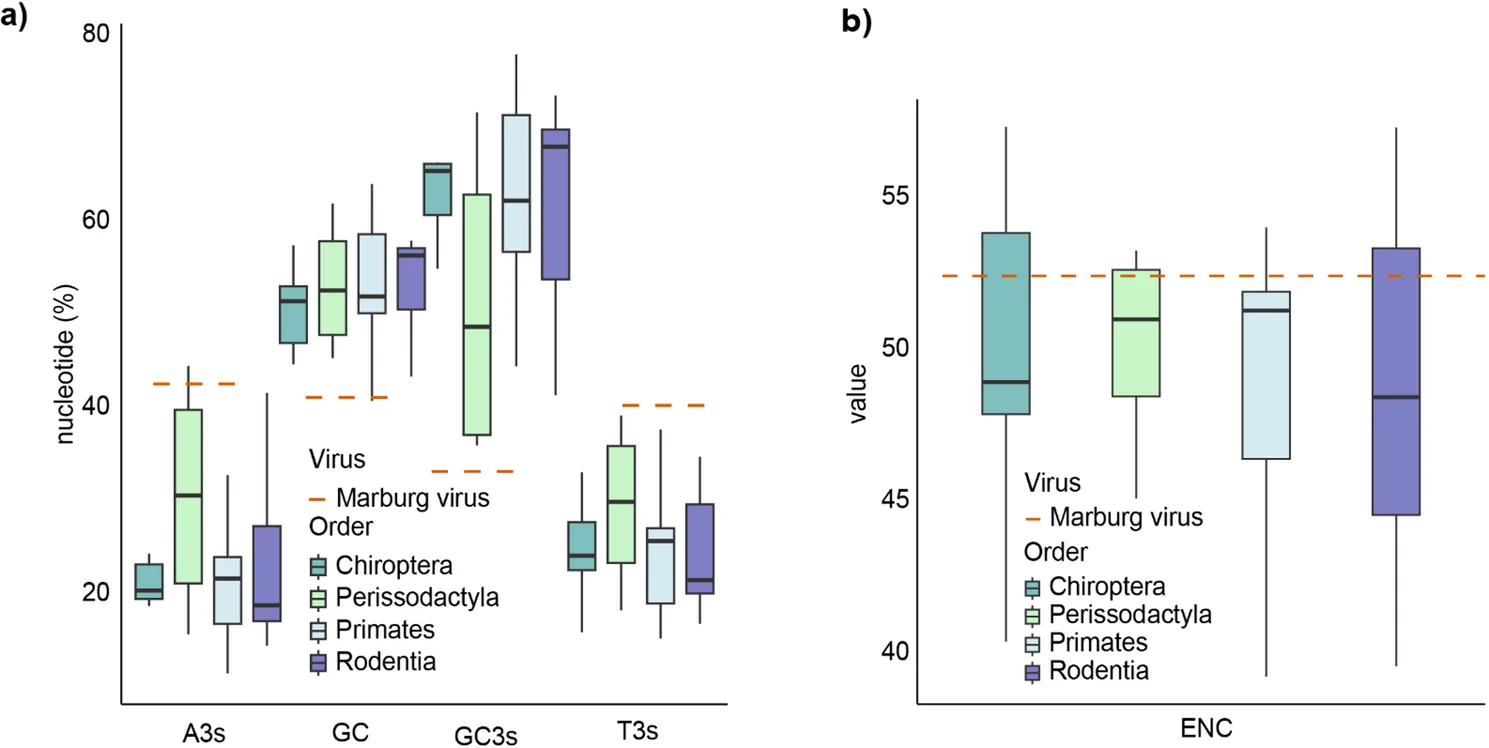
Nucleotide Composition and Codon Preferences of MARV and Mammals (**a**) GC Content and Third-Nucleotide Composition Percentages for Chiroptera, Perissodactyla, Primates, and Rodentia Codons (**b**) Effective Codon Number (ENC) for Chiroptera, Perissodactyla, Rodentia, and Primates The red dashed line denotes the Marburg virus. The four mammalian orders analyzed are represented by distinct colors as indicated in the legend. The figure presents a comparison of nucleotide composition similarities between the MARV and four animal orders, ranked from highest to lowest: Perissodactyla, Rodentia, Chiroptera, and Primates. The MARV sequence analyzed is: NC_024781.1

The genomic GC content of MARV is 40.2%, with a GC content at the third codon position ($\:{\mathrm{GC}}_{\mathrm{3s}}$) of 33.10%, reflecting a bias toward A/U nucleotides at this position. Regarding third-position nucleotide usage, MARV exhibits relative proximity to *Equus asinus* (37.40%) and *Equus quagga* (35.90%) within the order Perissodactyla, and *Gorilla gorilla gorilla* (44.40%) within order Primates. The effective number of codons (ENC) for MARV is 52.44, which closely approximates the values observed in *Rousettus aegyptiacus* (53.38), *Equus caballus* (52.43), *Equus asinus* (53.28), *Cryptomys hottentotus* (52.60) from Rodentia, and *Papio hamadryas* (51.92) from Primates (Fig. 5 and Additional file 1: Table S4).

### Evaluation of codon usage patterns based on ENC–GC3s analysis

**Fig. 6:**
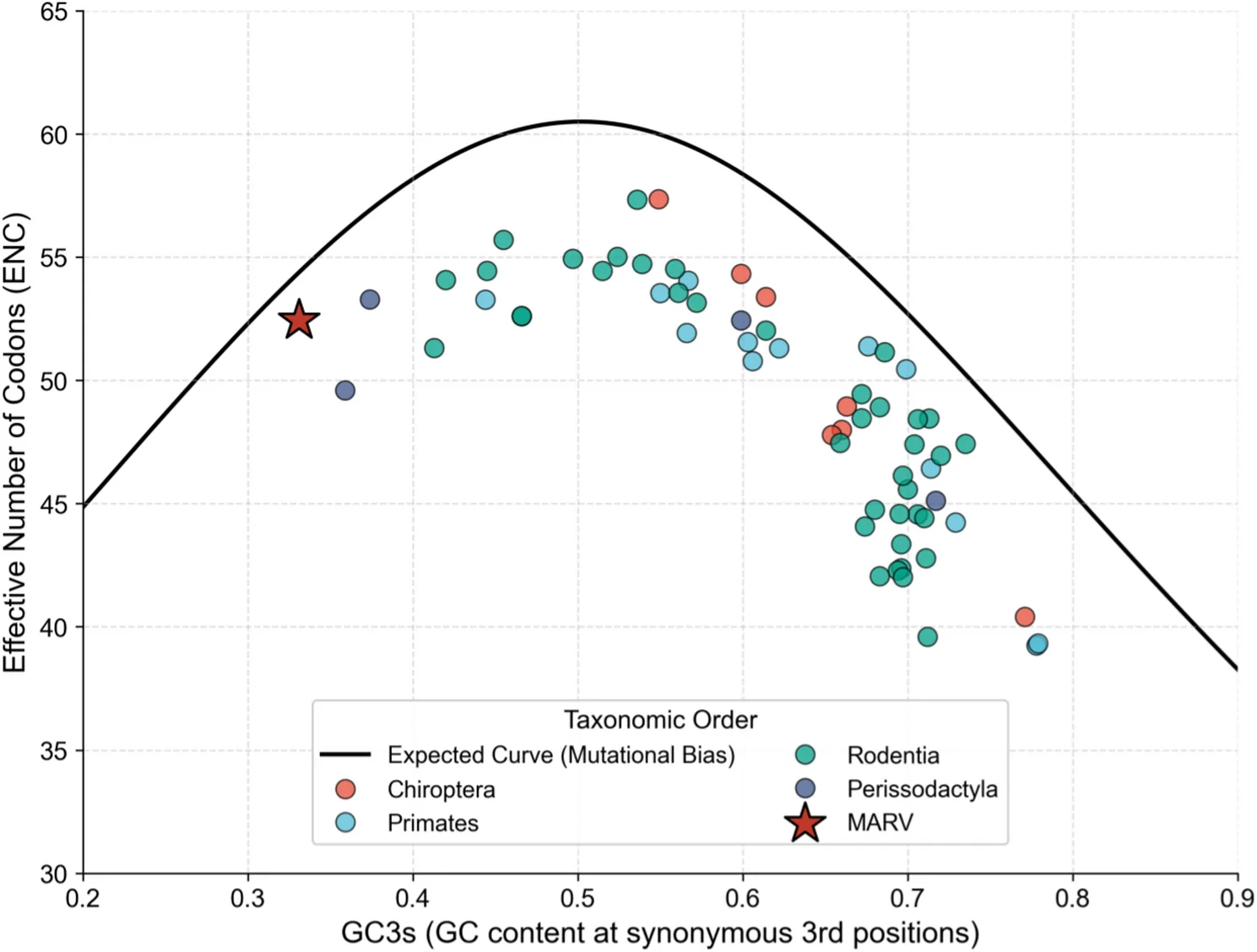
ENC–GC3s analysis of mutational and selective pressures in MARV and mammals codon usage. The solid black line denotes the theoretical ENC curve representing codon usage driven exclusively by mutation pressure. MARV (red star) is situated in the low GC3s region (0.331) and lies slightly below the expected curve, suggesting that its codon usage bias is primarily shaped by intrinsic genomic A/U mutation pressure rather than robust host-mediated translational selection. The distribution of mammalian hosts (colored dots) is broad: Rodentia (green) exhibit a degree of deviation from the null model, while Perissodactyla (dark blue) are the most proximal to MARV along the GC3s axis. Reference sequence: NC_024781.1

MARV data points are situated slightly below the theoretical mutation pressure curve and are characterized by low GC3s values. Distinct distribution patterns within the ENC–GC3s space were observed across the four mammalian orders: Rodentia predominantly exhibited high GC3s and low ENC values, while Primates and select Chiroptera were positioned within the intermediate range. Notably, Perissodactyla displayed the lowest GC3s ($\:\approx\:$ 0.35), exhibiting the highest proximity to MARV, with all representatives situated below the expected curve (Fig. 6 and Additional file 1: Table S5).

### Evaluation of suitability utilizing the Codon Adaptation Index (CAI)

**Fig. 7:**
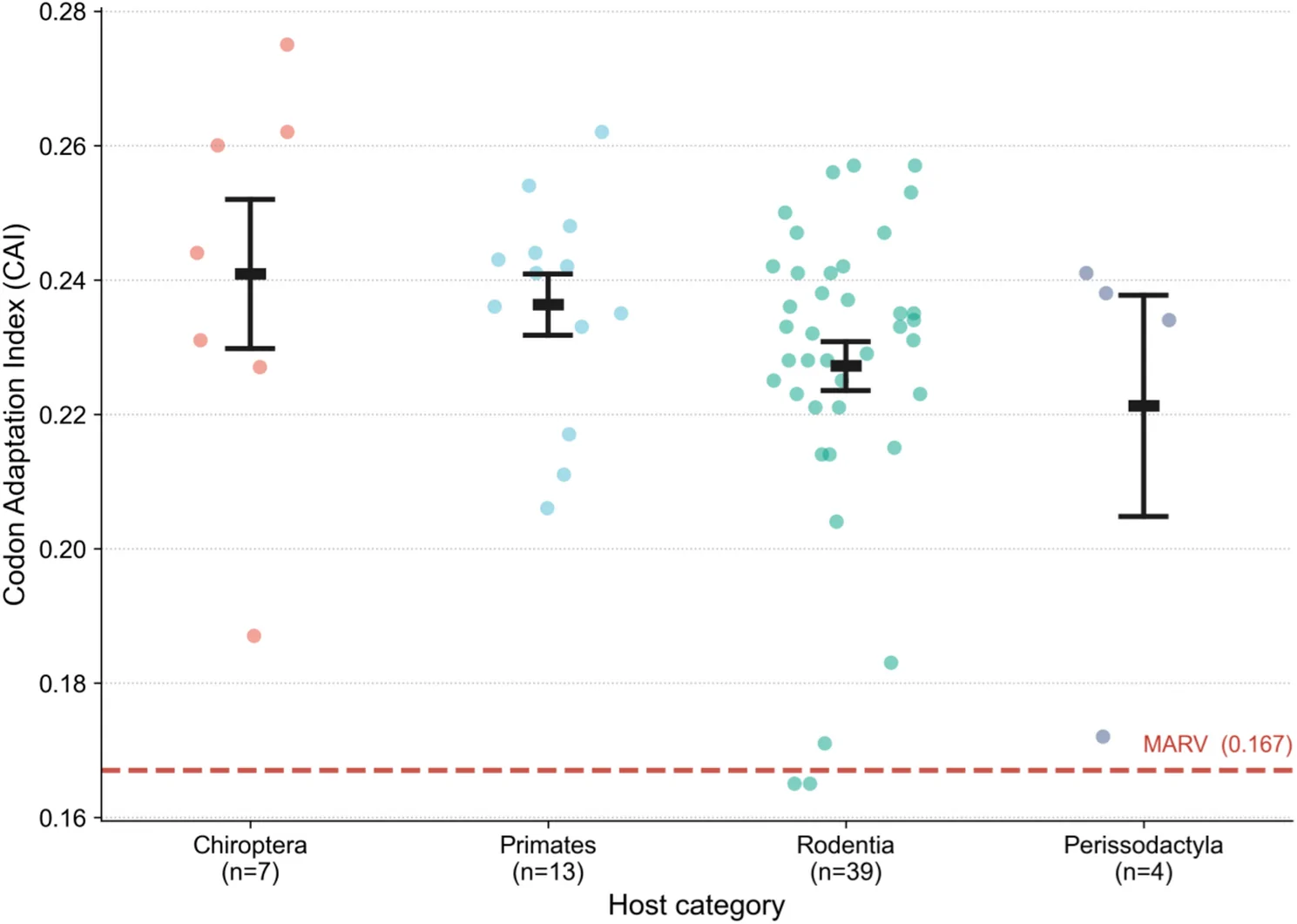
CAI of MARV relative to mammalian species Scatter plot illustrating the calculated CAI values for MARV across individual species; black bars indicate the mean "±" standard error (SEM). The red horizontal dotted line denotes the internal CAI baseline of MARV (0.167). Results demonstrate that MARV exhibits higher translational adaptability across all taxa compared to its own baseline. Notably, the order Chiroptera displays the highest mean CAI value. MARV reference sequence: NC_024781.1

Across the four evaluated groups, the order Chiroptera demonstrated the highest mean CAI ($\:\approx\:$ 0.241), followed by Primates ($\:\approx\:$ 0.236), Rodentia ($\:\approx\:$ 0.227), and Perissodactyla, which exhibited the lowest adaptation index ($\:\approx\:$ 0.221). Regarding specific species, those demonstrating CAI values most proximal to the MARV baseline (0.167) included *Cryptomys hottentotus pretoriae* (0.165), *Cryptomys hottentotus* (0.165), *Rattus norvegicus* (0.171), and *Equus quagga* (0.172) (Fig. 7).

### Evaluation of dinucleotide usage profiles and CpG suppression relative to the null model

**Fig. 8:**
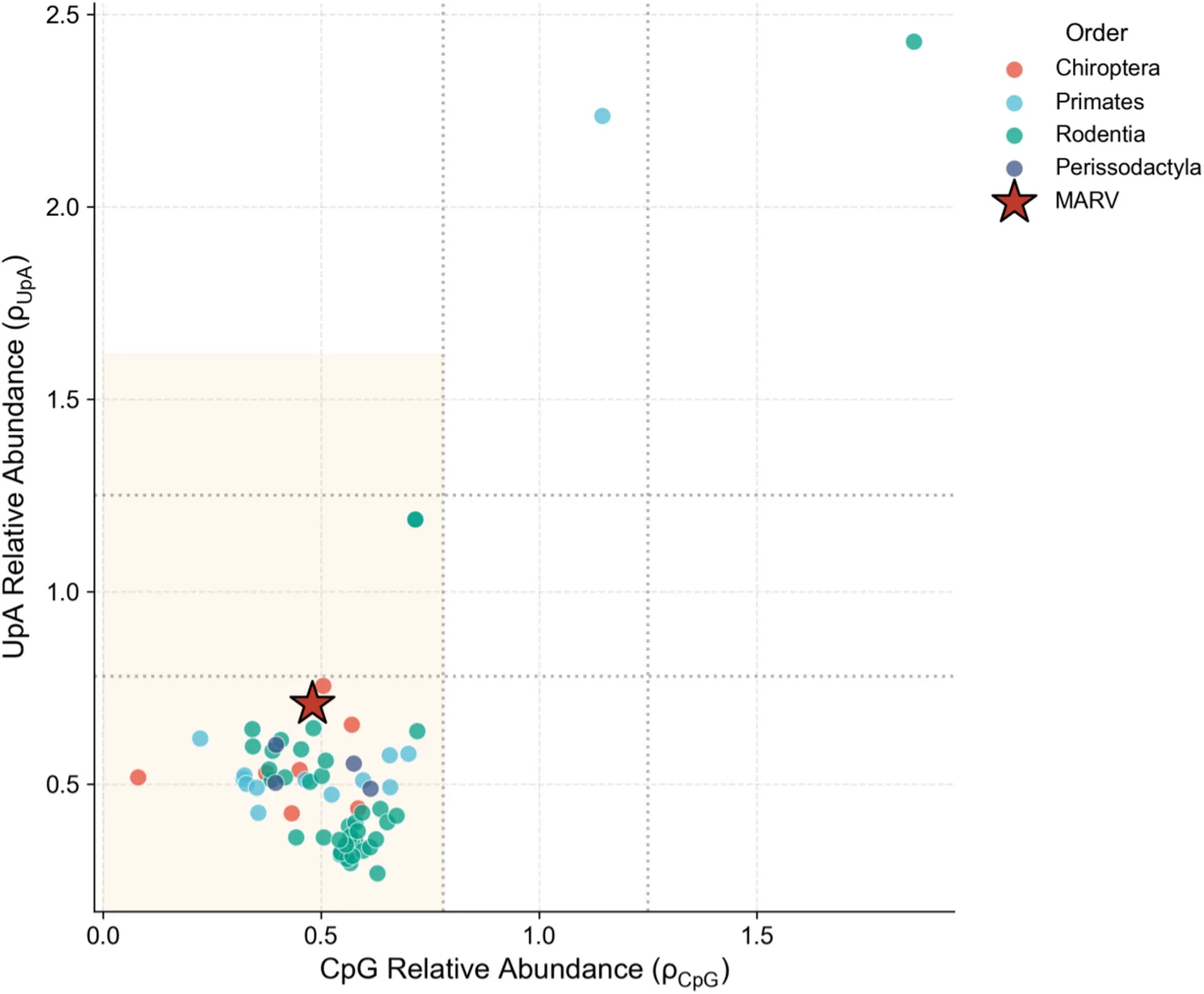
Relative abundance profiles of CpG and UpA dinucleotides in MARV and mammals. Scatter plot illustrating dinucleotide usage preferences across species, with MARV denoted by red stars. The thick dashed line represents the theoretical threshold for dinucleotide suppression (relative abundance ≤ 0.78), a value typically indicating significant compositional avoidance. MARV is positioned well within this region of pronounced suppression. MARV reference sequence: NC_024781.1

MARV, consistent with the majority of mammals, is localized within the lower-left quadrant characterized by the dual suppression of CpG and UpA, with MARV positioned at $\rho_{\mathrm{CpG}}\approx$0.48 and $\rho_{\mathrm{UpA}}\approx$ 0.75. Most mammalian species are concentrated within the ranges of $\rho_{\mathrm{CpG}}$ 0.35–0.70 and $\rho_{\mathrm{UpA}}$ 0.25–0.70; among these, members of the order Perissodactyla exhibit CpG levels most proximal to those of MARV. Notably, one species within the order Chiroptera displays $\rho_{\mathrm{CpG}}\approx$ 0.08, representing the most pronounced CpG suppression among the evaluated taxa (Fig. 8).

The observed $\:{\rho\:}_{CpG}$ value of 0.48 for MARV is significantly lower than the stochastic null distribution. All four mammalian orders exhibited significantly reduced mean $\:{\rho\:}_{CpG}$ values. When ranked by increasing $\:{\rho\:}_{CpG}$, the sequence was: Chiroptera, Perissodactyla, Primates, and Rodentia. Notably, the mean values for Perissodactyla and Primates were highly comparable to those of MARV (difference $\:\approx\:$ 0.02–0.03) (Fig. S2).

To provide an integrated overview of the analytical framework and the principal observations across these complementary approaches, the study design and major molecular findings are summarised in Table 1.

**Table 1:** Summary of the main analytical approaches and key molecular characteristics

Analytical Dimension	Methodology	Key Molecular Observations	Evidence Class
Sequence similarity	BLAST, null-model	A few fragments matched Rodentia and Perissodactyla sequences more frequently than expected by chance.	Local sequence signals
Codon composition	CLR-PCA	MARV was separated from the overall distribution of mammals, whereas Perissodactyla and some Rodentia were relatively close.	Overall compositional characteristics
Codon bias distance	RSCU distance	Some Perissodactyla and Rodentia species were closer to MARV.	Numerical proximity
Nucleotide composition	GC, GC3, ENC	Perissodactyla showed relative proximity to MARV in terms of GC3 and ENC.	Compositional characteristics
Bias mechanism	ENC-GC3s	MARV was located in the mutation pressure-dominated region, and Perissodactyla was closely aligned with it in the low-GC3s region.	Bias patterns
Translational fitness	CAI	Differences were observed across taxonomic groups, and some species were numerically close to MARV.	Relative fitness
Dinucleotide composition	CpG/UpA	MARV shared a CpG suppression pattern with multiple groups, and Perissodactyla was relatively close to Primates.	Compositional characteristics

## Discussion

While *Rousettus aegyptiacus* remains recognized as the principal natural reservoir of Marburg virus (MARV), reliance on a solitary bat-host model is increasingly challenged by the virus’s complex epidemiological and ecological profiles [15–17]. Recent outbreak characteristics heavily imply more intricate regional transmission dynamics and broader host-ecological networks than previously assumed. Specifically, the strain responsible for the 2023 outbreak in Equatorial Guinea exhibited substantial genetic heterogeneity compared to historical isolates [18, 19]; several spillover events, notably in Tanzania, occurred in the absence of discernible bat contact [20]; and the modest viral loads and restricted shedding durations documented in fruit bats fail to fully resolve the mechanisms of spillover propagation [21, 22]. These observations suggest that MARV persistence and transmission are governed by networks far more complex than those accommodated by a single-reservoir model. By utilizing a multi-pronged computational genomics framework, this study systematically evaluated the molecular compatibility between MARV and various mammalian orders across sequence similarity, codon usage profiles, composition indices, and dinucleotide biases. Our parallel methodologies yielded highly congruent results, capturing distinct signals of molecular proximity within Perissodactyla and select Rodentia groups. Remarkably, *Equus asinus* displayed the shortest RSCU distance (0.25) to MARV, while the ENC of *Equus caballus* (52.43) was virtually identical to the viral value (52.44). Similar translational compatibility was mirrored in rodent species, such as *Cryptomys hottentotus*. Collectively, these cross-dimensional findings reveal that MARV possesses significant genomic and translational congruence with specific non-chiropteran mammalian taxa. These insights offer invaluable pathways for targeted ecological surveillance and for modeling the multi-host dynamics of zoonotic transmission.

Divergent sequence alignments revealed that several MARV genomic fragments possess a stochastically enriched frequency of sequence matches with the orders Perissodactyla and Rodentia. These alignment signatures represent promising exploratory markers; their presence in key functional protein-coding domains (specifically the NP and GP genes) highlights genomic regions that merit targeted investigation. These findings are further reinforced by our characterization of codon preferences: *Equus asinus* exhibited the lowest RSCU distance to MARV, whereas the ENC of *Equus caballus* closely approximated that of the viral genome. This cross-clade similarity suggests that MARV’s nucleotide composition shares a notable quantitative correspondence with specific mammalian lineages, potentially reflecting genomic convergence driven by mutational pressure and shared molecular constraints during viral evolution [23–26].

To determine whether these compositional congruencies are reflected within the broader codon space, this study combined an *ENC--GC3s* plot analysis to evaluate the relationship between codon bias and GC content at the third codon position. The resulting topology demonstrates that MARV is positioned adjacent to the theoretical expectation curve, indicating that its codon usage is primarily shaped by genomic compositional bias (mutational pressure) rather than intense host-mediated selective pressures [23, 25, 26]. This pattern displays a distinct compositional convergence with Perissodactyla, particularly in the low-$\:{\mathrm{GC}}_{\mathrm{3s}}$ and high-ENC regions where the two groups exhibit local proximity to the virus. In contrast, rodents reside within a higher $\:{\mathrm{GC}}_{\mathrm{3s}}$ range, reflecting a different genomic background. This molecular parallelism suggests that MARV and perissodactyls may share comparable nucleotide compositional constraints; physiological variables such as metabolic rate, DNA repair efficiency, or oxidative stress tolerance may collectively contribute to driving this convergent molecular adaptation.

Our assessment of the Codon Adaptation Index (CAI) further resolves variations in translational adaptation. While Chiroptera demonstrated superior translational efficiency—marked by elevated CAI levels (≈ 0.187–0.23) that align with their canonical reservoir role—members of Perissodactyla and Rodentia yielded values closely approximating or marginally surpassing the viral baseline (MARV CAI = 0.167). This indicates a moderate level of molecular proximity. This stratification implies that MARV’s translational fitness is highly customized across different host environments, illustrating that the molecular interfaces governing host-pathogen associations are far too intricate to be simplified into a single, isolated parameter [23, 27, 28].

Delineation of dinucleotide frequencies revealed marked CpG and UpA depletion in MARV, with CpG levels suppressed significantly beyond stochastic expectations of the null model, aligning with established vertebrate-associated viral signatures [29–32]. This pattern implies that MARV’s genomic composition is modulated by systemic, host-mediated selective pressures—likely related to innate immune recognition—rather than host-specific replication optimization. Underrepresented CpG and UpA motifs may thus act as a generic determinant of host-pathogen molecular compatibility that operates independently of host identity. Notably, Perissodactyla and Primates demonstrated striking quantitative alignment with MARV in their CpG profiles (difference *≤* 0.1), whereas Rodentia exhibited a marked divergence. This compositional similarity implies that Perissodactyla may impose or share comparable immunogenic constraints with MARV at the dinucleotide interface. Nevertheless, the precise mechanisms driving this evolutionary convergence require further elucidation through the integration of host restriction factor assays and in vitro replication dynamics [32–36].

Synthesizing these multidimensional genomic insights, MARV exhibits a remarkably stable yet broadly congruent genomic composition profile rather than a rigidly specialized molecular signature, manifesting as varying degrees of localized and global homology across diverse mammalian clades. Rather than pointing to a singular, evolutionary optimized host, this molecular plasticity suggests a potential capacity for multi-host compatibility, with members of Perissodactyla and specific Rodentia lineages consistently demonstrating close spatial and molecular proximity across multiple independent genomic indices. While *Rousettus aegyptiacus* remains the canonical natural reservoir supported by the most robust empirical evidence, Perissodactyla and select rodents emerge as compelling candidate clades that warrant targeted investigation in future surveillance [37]. Ultimately, this high-dimensional molecular comparison provides crucial, complementary genomic evidence to assess the potential risk of cross-species transmission cascades and guide systematic host-screening efforts.

While our study provides a comprehensive computational framework, several inherent limitations must be acknowledged. First, because our findings rely strictly on bioinformatics profiling, the observed alignments in codon usage and dinucleotide composition represent a baseline of theoretical molecular affinity rather than biological susceptibility; they do not establish definitive viral entry, replication competency, or transmission dynamics. Second, some alignment profiles were restricted to relatively short sequence fragments, and public CDS databases remain subject to significant biases in both annotation completeness and taxonomic representation. Thus, the inclusion of certain non-native African species should be viewed as an index of evolutionary compatibility at the family or order level, rather than an indicator of direct local ecological overlap. Ultimately, pending validation through in vitro virological assays, seroprevalence studies, and active field-based surveillance, these results should be regarded as speculative, hypothesis-generating clues intended to direct future research priorities rather than a definitive resolution of MARV’s natural host spectrum.

From an ecological standpoint, this framework substantively complements the established epidemiological paradigm of MARV transmission. While *Rousettus aegyptiacus* remains the definitive natural reservoir supported by the most robust empirical evidence [15, 16], our multidimensional genomic profiling suggests that alternative mammalian cohorts—specifically domesticated perissodactyls (such as *Equus asinus* and *Equus quagga*) situated at the anthropogenic interface, alongside ubiquitous rodent populations—represent plausible candidates for targeted monitoring and validation. This multi-taxa perspective offers novel mechanistic hypotheses to reconcile the enigmatic geographical distribution patterns, cryptic exposure histories, and unresolved transmission pathways observed in atypical outbreaks. Concurrently, it delineates high-priority targets for subsequent active field surveillance, seroprevalence screening, and functional virological validation.

## Conclusions

MARV exhibits heterogeneous degrees of genomic similarity across diverse host clades regarding molecular composition, codon usage, and translational fitness metrics; among these, Perissodactyla and select rodent lineages demonstrate consistent proximity across multiple parameters, whilst Chiroptera stands out in translational fitness indicators. The integrated results of these analyses collectively delineate the patterns of molecular associations between MARV and various mammalian groups. From a computational biology perspective, this study has identified several candidate host groups warranting targeted investigation, providing high-priority research leads for subsequent field monitoring, serological surveys, virological testing, and experimental validation within a One Health framework.

## Supplementary Information

Supplementary Material 1.

## Data Availability

The datasets analyzed during the current study are publicly available in the NCBI Nucleotide database.

## Abbreviations

MARV: Marburg virus
CDS: Coding DNA Sequences
nt: Nucleotide
CAI: Codon Adaptation Index
RSCU: Relative Synonymous Codon Usage
ENC: Effective Number of Codons
GC3s: GC content at the third synonymous codon position
CpG: Cytosine-phosphate-Guanine dinucleotide
UpA: Uridine-phosphate-Adenosine dinucleotide
ZAP: Zinc finger antiviral protein
TLR9: Toll-like receptor 9
NP: Nucleoprotein
GP: Glycoprotein
BLAST: Basic Local Alignment Search Tool
