# Supplementary material for: Divergent host adaptation in Marburg virus: a hypothesis-generating framework for prioritizing non-traditional reservoirs
Source: BMC Genomics. 2026 Aug 6;27:769. doi: 10.1186/s12864-026-13256-y (PMC13589372; doi:10.1186/s12864-026-13256-y)
Supplement: Supplementary file 1 [file 12864_2026_13256_MOESM1_ESM.docx]

Additional file 1

**Divergent host adaptation in Marburg virus: a hypothesis-generating framework for prioritizing non-traditional reservoirs**

Qi Chen,^1†^ Jingjing Hu,^1,2†^ Siru Hu,^3†^ Yuxi Wang,^3^ Weijie Chen,^3,4^ Shuiping Lu,^3^ Jiaxu Chen,^1^ Sijia Zhuang,^3^ Zhong Sun,^5,6^ Karuppiah Thilakavathy,^6^ Lufang Jiang,^3*^ Chenglong Xiong^3*^

Fig. S1: Phylogenetic relationships between specific genomic segments of the MARV and mammals.

Fig. S2: Evaluation of evolutionary selection pressure on CpG dinucleotides in MARV utilizing a null model.

Table S1:Comparison of viral fragment match frequency against random null sequences and empirical P-values.

Table S2: Table S2: Homology analysis results of Marburg virus genome fragments

Table S3: Average Manhattan Distance of Relative Synonymous Codon Usage Between 63 Species and Marburg Virus.

Table S4: Codon Base Composition of Marburg Virus and 63 Animal Species

Table S5: Codon Usage Parameters of Marburg Virus and 63 Animal Species.

Fig. S1: Phylogenetic relationships between specific genomic segments of the MARV and mammals.

The tree illustrates the associations between MARVfragments ( 901–1200 nt and 18601–18900 nt ) and sequences (≥ 50 nt) from Rodentia and Perissodactyla identified through distant-homology BLAST searches. Red branches denote two distinct MARV lineages: the Ravn and Musoke strains.

Fig. S2: Evaluation of evolutionary selection pressure on CpG dinucleotides in MARV utilizing a null model.

Expected CpG distribution (grey histogram/density curve) was generated by 1,000 sequence randomizations (null model mean = 0.96). The observed MARV CpG abundance (0.48) is represented by the red solid line. Dashed and dotted lines denote the mean CpG relative abundances for the mammalian orders. Reference sequence: NC_024781.1.

Table S1:Comparison of viral fragment match frequency against random null sequences and empirical P-values.

| Virus Fragment | Target Taxon | Observed Hits | Null Average, n=20 | Null Max | EmpiricalP-value |
| --- | --- | --- | --- | --- | --- |
| 1-Hit | Rodentia | 1 | 0 | 0 | < 0.05 |
| 2-Hit | Rodentia | 10 | 0 | 0 | < 0.05 |
| 3-Hit | Rodentia | 3 | 0.1 | 2 | < 0.05 |
| 4-Hit | Perissodactyla | 12 | 0 | 0 | < 0.05 |

Table S2: Homology analysis results of Marburg virus genome fragments

| Fragment name | species name | Sequence ID |
| --- | --- | --- |
| 1987_1-300 | *Agathobacter rectalis* | CP143947.1 |
|  | *Peromyscus eremicus* | XM_059276182.1 |
|  | *Boldia erythrosiphon* | NC_034776.1 |
| 1987_901-1200 | *Microtus pennsylvanicus* | KM189811.1 |
|  | *Peromyscus eremicus* | XR_009380245.1 |
|  | *Peromyscus californicus* | XR_008221635.1 |
|  | *Arvicola amphibius* | XM_038346334.1 |
|  | *Microtus oregoni* | XM_041648953.1 |
|  | *Microtus ochrogaster* | XM_005344680.2 |
| 1987_9901-10200 | *Strongyloides papillosus* | LM525680.1 |
|  | Mouse | AL844146.2 |
|  | *Mus musculus* | AC079439.26 |
|  | *Drosophila miranda* | XM_017290151.2 |
| 1987_18601-18900 | *Lutzomyia longipalpis* | XM_055821203.1 |
|  | *Oppiella nova* | OC936490.1 |
|  | *Candidatus Shikimatogenerans bostrichidophilus* | CP099825.1 |
|  | *Photobacterium phosphoreum* | OX460910.1 |
|  | *M.truncatula* | CU468264.8 |
|  | *Equus asinus* | XM_014857017.2 |
|  | *Equus przewalskii* | XM_008537398.1 |
|  | *Candidatus Carsonella ruddii* | CP092145.1 |
|  | *Equus quagga* | XM_046665517.1 |
|  | *Candidatus Carsonella ruddii* | CP102595.1 |
|  | *Equus caballus* | OZ014259.2 |
|  | *Lucilia sericata* | XM_037960215.1 |
|  | *Candidatus Stammera* | CP043980.1 |
|  | *Candidatus Phytoplasma* | CP097583.1 |

Table S3: Average Manhattan Distance of Relative Synonymous Codon Usage Between 63 Species and Marburg Virus.

| species name | DSE |
| --- | --- |
| *Coleura afra* | 0.54 |
| *Eidolon helvum* | 0.66 |
| *Hipposideros caffer* | 0.71 |
| *Myotis daubentonii* | 0.67 |
| *Pipistrellus sp. DR-2011* | 0.89 |
| *Rhinolophus landeri* | 0.65 |
| *Rousettus aegyptiacus* | 0.69 |
| *Cercopithecus cephus* | 0.85 |
| *Cercopithecus neglectus* | 0.59 |
| *Chlorocebus aethiops* | 0.78 |
| *Chlorocebus pygerythrus* | 0.94 |
| *Chlorocebus sabaeus* | 0.81 |
| *Chlorocebus tantalus* | 0.56 |
| *Colobus guereza* | 0.60 |
| *Gorilla gorilla gorilla* | 0.29 |
| *Macaca fascicularis* | 0.49 |
| *Macaca mulatta* | 0.67 |
| *Pan troglodytes* | 0.94 |
| *Papio Anubis* | 0.48 |
| *Papio hamadryas* | 0.52 |
| *Acomys johannis* | 0.86 |
| *Apodemus flavicollis* | 0.80 |
| *Arvicanthis niloticus* | 0.79 |
| *Bandicota indica* | 0.79 |
| *Bandicota savilei* | 0.75 |
| *Bathyergus janetta* | 0.38 |
| *Bathyergus suillus* | 0.48 |
| *Berylmys berdmorei* | 0.80 |
| *Berylmys bowersi* | 0.80 |
| *Cryptomys hottentotus* | 0.35 |
| *Cryptomys hottentotus pretoriae* | 0.35 |
| *Fukomys damarensis* | 0.59 |
| *Georychus capensis* | 0.29 |
| *Hapalomys delacouri* | 0.73 |
| *Hystrix africaeaustralis* | 0.42 |
| *Lemniscomys zebra* | 0.83 |
| *Leopoldamys edwardsi* | 0.84 |
| *Mastomys erythroleucus* | 0.84 |
| *Mastomys kollmannspergeri* | 0.84 |
| *Mastomys natalensisv* | 0.84 |
| *Maxomys surifer* | 0.80 |
| *Mus caroli* | 0.82 |
| *Mus cervicolor* | 0.80 |
| *Mus cookii* | 0.82 |
| *Mus mattheyi* | 0.77 |
| *Mus musculus* | 0.43 |
| *Niviventer fulvescens* | 0.79 |
| *Praomys sp. DB-2012* | 0.85 |
| *Rattus exulans* | 0.78 |
| *Rattus losea* | 0.79 |
| *Rattus norvegicus* | 0.31 |
| *Rattus tanezumi* | 0.66 |
| *Thryonomys swinderianus* | 0.83 |
| *Peromyscus eremicus* | 0.34 |
| *Microtus pennsylvanicus* | 0.50 |
| *Peromyscus californicus* | 0.31 |
| *Arvicola amphibius* | 0.38 |
| *Microtus oregoni* | 0.49 |
| *Microtus ochrogaster* | 0.40 |
| *Equus asinus* | 0.25 |
| *Equus przewalskii* | 0.79 |
| *Equus caballus* | 0.57 |
| *Equus quagga* | 0.29 |

Table S4: Codon Base Composition of Marburg Virus and 63 Animal Species

|  | GC | A3s | T3s | G3s | C3s |
| --- | --- | --- | --- | --- | --- |
| Marburg virus | 0.402 | 0.425 | 0.4105 | 0.2108 | 0.2187 |
| *Coleura afra* | 0.475 | 0.2424 | 0.3302 | 0.3092 | 0.4093 |
| *Eidolon helvum* | 0.52 | 0.1865 | 0.2404 | 0.4168 | 0.4462 |
| *Hipposideros caffer* | 0.54 | 0.2032 | 0.2155 | 0.405 | 0.4613 |
| *Myotis daubentonii* | 0.514 | 0.2015 | 0.2345 | 0.4174 | 0.4477 |
| *Pipistrellus sp. DR-2011* | 0.574 | 0.1207 | 0.1583 | 0.4556 | 0.5324 |
| *Rhinolophus landeri* | 0.446 | 0.2203 | 0.3071 | 0.4299 | 0.4016 |
| *Rousettus aegyptiacus* | 0.463 | 0.2419 | 0.246 | 0.3874 | 0.4286 |
| *Cercopithecus cephus* | 0.57 | 0.1806 | 0.1901 | 0.4815 | 0.4085 |
| *Cercopithecus neglectus* | 0.473 | 0.224 | 0.26 | 0.3394 | 0.44 |
| *Chlorocebus aethiops* | 0.586 | 0.1671 | 0.1892 | 0.4626 | 0.4484 |
| *Chlorocebus pygerythrus* | 0.64 | 0.1153 | 0.1516 | 0.4821 | 0.4695 |
| *Chlorocebus sabaeus* | 0.604 | 0.1458 | 0.1829 | 0.4472 | 0.454 |
| *Chlorocebus tantalus* | 0.514 | 0.2223 | 0.2564 | 0.4378 | 0.3708 |
| *Colobus guereza* | 0.475 | 0.216 | 0.27 | 0.3379 | 0.4367 |
| *Gorilla gorilla gorilla* | 0.407 | 0.327 | 0.376 | 0.2651 | 0.3196 |
| *Macaca fascicularis* | 0.509 | 0.239 | 0.3075 | 0.3876 | 0.3578 |
| *Macaca mulatta* | 0.544 | 0.1947 | 0.2053 | 0.4678 | 0.3842 |
| *Pan troglodytes* | 0.637 | 0.1141 | 0.152 | 0.4742 | 0.4853 |
| *Papio Anubis* | 0.501 | 0.2762 | 0.2701 | 0.2708 | 0.4123 |
| *Papio hamadryas* | 0.519 | 0.2595 | 0.2752 | 0.3498 | 0.3624 |
| *Acomys johannis* | 0.575 | 0.1987 | 0.1677 | 0.3562 | 0.5226 |
| *Apodemus flavicollis* | 0.571 | 0.1788 | 0.1818 | 0.4143 | 0.4805 |
| *Arvicanthis niloticus* | 0.565 | 0.1776 | 0.2117 | 0.3759 | 0.4886 |
| *Bandicota indica* | 0.563 | 0.1683 | 0.2226 | 0.3763 | 0.4806 |
| *Bandicota savilei* | 0.561 | 0.1753 | 0.2226 | 0.3669 | 0.4817 |
| *Bathyergus janetta* | 0.441 | 0.321 | 0.3067 | 0.2658 | 0.3716 |
| *Bathyergus suillus* | 0.498 | 0.2629 | 0.2932 | 0.3443 | 0.3851 |
| *Berylmys berdmorei* | 0.564 | 0.1883 | 0.2 | 0.3636 | 0.4968 |
| *Berylmys bowersi* | 0.567 | 0.1871 | 0.2129 | 0.3586 | 0.4839 |
| *Cryptomys hottentotus* | 0.476 | 0.3626 | 0.2692 | 0.3027 | 0.2635 |
| *Cryptomys hottentotus pretoriae* | 0.476 | 0.3629 | 0.2686 | 0.3029 | 0.2637 |
| *Fukomys damarensis* | 0.521 | 0.226 | 0.2615 | 0.3619 | 0.436 |
| *Georychus capensis* | 0.433 | 0.351 | 0.3333 | 0.2637 | 0.3285 |
| *Hapalomys delacouri* | 0.562 | 0.1842 | 0.2179 | 0.3759 | 0.4744 |
| *Hystrix africaeaustralis* | 0.45 | 0.277 | 0.3017 | 0.3179 | 0.3796 |
| *Lemniscomys zebra* | 0.568 | 0.1579 | 0.2143 | 0.4029 | 0.487 |
| *Leopoldamys edwardsi* | 0.564 | 0.1871 | 0.1859 | 0.3681 | 0.5064 |
| *Mastomys erythroleucus* | 0.577 | 0.1503 | 0.2052 | 0.3916 | 0.5081 |
| *Mastomys kollmannspergeri* | 0.579 | 0.1438 | 0.2026 | 0.3986 | 0.5163 |
| *Mastomys natalensisv* | 0.572 | 0.1513 | 0.2039 | 0.3873 | 0.5197 |
| *Maxomys surifer* | 0.571 | 0.183 | 0.1889 | 0.3732 | 0.5049 |
| *Mus caroli* | 0.575 | 0.1678 | 0.1867 | 0.3841 | 0.5133 |
| *Mus cervicolor* | 0.575 | 0.1711 | 0.1908 | 0.3759 | 0.5132 |
| *Mus cookii* | 0.576 | 0.1678 | 0.1839 | 0.3841 | 0.5135 |
| *Mus mattheyi* | 0.571 | 0.1824 | 0.1769 | 0.3696 | 0.517 |
| *Mus musculus* | 0.51 | 0.2675 | 0.3056 | 0.328 | 0.3607 |
| *Niviventer fulvescens* | 0.563 | 0.1765 | 0.2065 | 0.3759 | 0.4903 |
| *Praomys sp. DB-2012* | 0.574 | 0.1711 | 0.2027 | 0.3732 | 0.5068 |
| *Rattus exulans* | 0.57 | 0.1691 | 0.2054 | 0.3726 | 0.5014 |
| *Rattus losea* | 0.571 | 0.1694 | 0.2 | 0.3798 | 0.4973 |
| *Rattus norvegicus* | 0.549 | 0.36 | 0.3374 | 0.2057 | 0.3009 |
| *Rattus tanezumi* | 0.539 | 0.1931 | 0.224 | 0.3721 | 0.4609 |
| *Thryonomys swinderianus* | 0.53 | 0.1599 | 0.1675 | 0.4263 | 0.5129 |
| *Peromyscus eremicus* | 0.500 | 0.4154 | 0.3196 | 0.1864 | 0.3284 |
| *Microtus pennsylvanicus* | 0.530 | 0.207 | 0.3038 | 0.2985 | 0.3824 |
| *Peromyscus californicus* | 0.474 | 0.3364 | 0.3467 | 0.2422 | 0.3216 |
| *Arvicola amphibius* | 0.499 | 0.3005 | 0.3063 | 0.3272 | 0.3348 |
| *Microtus oregoni* | 0.524 | 0.2614 | 0.2693 | 0.3564 | 0.3725 |
| *Microtus ochrogaster* | 0.495 | 0.3008 | 0.2981 | 0.3355 | 0.3445 |
| *Equus asinus* | 0.453 | 0.3816 | 0.391 | 0.209 | 0.2682 |
| *Equus przewalskii* | 0.619 | 0.1562 | 0.1819 | 0.4254 | 0.4458 |
| *Equus caballus* | 0.565 | 0.2289 | 0.2495 | 0.3247 | 0.4051 |
| *Equus quagga* | 0.486 | 0.4444 | 0.3473 | 0.176 | 0.2698 |

Table S5: Codon Usage Parameters of Marburg Virus and 63 Animal Species.

|  | CAI | CBI | Fop | ENC | GC3s |
| --- | --- | --- | --- | --- | --- |
| Marburg virus | 0.167 | -0.122 | 0.344 | 52.44 | 0.331 |
| *Coleura afra* | 0.262 | 0.029 | 0.439 | 57.35 | 0.549 |
| *Eidolon helvum* | 0.231 | 0.087 | 0.466 | 47.99 | 0.660 |
| *Hipposideros caffer* | 0.275 | 0.143 | 0.504 | 48.94 | 0.663 |
| *Myotis daubentonii* | 0.244 | 0.106 | 0.480 | 47.78 | 0.654 |
| *Pipistrellus sp. DR-2011* | 0.260 | 0.173 | 0.516 | 40.40 | 0.771 |
| *Rhinolophus landeri* | 0.227 | 0.079 | 0.463 | 54.32 | 0.599 |
| *Rousettus aegyptiacus* | 0.187 | 0.030 | 0.418 | 53.38 | 0.614 |
| *Cercopithecus cephus* | 0.242 | 0.077 | 0.449 | 50.45 | 0.699 |
| *Cercopithecus neglectus* | 0.233 | 0.103 | 0.468 | 50.78 | 0.606 |
| *Chlorocebus aethiops* | 0.254 | 0.132 | 0.499 | 46.42 | 0.714 |
| *Chlorocebus pygerythrus* | 0.243 | 0.171 | 0.520 | 39.24 | 0.778 |
| *Chlorocebus sabaeus* | 0.241 | 0.144 | 0.502 | 44.23 | 0.729 |
| *Chlorocebus tantalus* | 0.244 | 0.079 | 0.463 | 51.30 | 0.622 |
| *Colobus guereza* | 0.236 | 0.119 | 0.476 | 51.55 | 0.603 |
| *Gorilla gorilla gorilla* | 0.206 | 0.018 | 0.418 | 53.27 | 0.444 |
| *Macaca fascicularis* | 0.248 | 0.056 | 0.447 | 54.04 | 0.567 |
| *Macaca mulatta* | 0.235 | 0.104 | 0.472 | 51.38 | 0.676 |
| *Pan troglodytes* | 0.262 | 0.190 | 0.533 | 39.32 | 0.779 |
| *Papio Anubis* | 0.217 | 0.059 | 0.448 | 53.54 | 0.550 |
| *Papio hamadryas* | 0.211 | 0.002 | 0.410 | 51.92 | 0.566 |
| *Acomys johannis* | 0.238 | 0.145 | 0.489 | 45.57 | 0.700 |
| *Apodemus flavicollis* | 0.235 | 0.087 | 0.455 | 44.56 | 0.706 |
| *Arvicanthis niloticus* | 0.229 | 0.090 | 0.453 | 42.05 | 0.683 |
| *Bandicota indica* | 0.225 | 0.086 | 0.452 | 44.75 | 0.680 |
| *Bandicota savilei* | 0.221 | 0.090 | 0.454 | 44.07 | 0.674 |
| *Bathyergus janetta* | 0.204 | 0.016 | 0.424 | 54.93 | 0.497 |
| *Bathyergus suillus* | 0.233 | 0.036 | 0.440 | 54.52 | 0.559 |
| *Berylmys berdmorei* | 0.228 | 0.138 | 0.481 | 48.91 | 0.683 |
| *Berylmys bowersi* | 0.228 | 0.119 | 0.471 | 48.46 | 0.672 |
| *Cryptomys hottentotus* | 0.165 | -0.028 | 0.392 | 52.60 | 0.466 |
| *Cryptomys hottentotus pretoriae* | 0.165 | -0.028 | 0.392 | 52.61 | 0.466 |
| *Fukomys damarensis* | 0.247 | 0.093 | 0.474 | 52.02 | 0.614 |
| *Georychus capensis* | 0.215 | 0.021 | 0.428 | 55.70 | 0.455 |
| *Hapalomys delacouri* | 0.214 | 0.105 | 0.466 | 49.44 | 0.672 |
| *Hystrix africaeaustralis* | 0.214 | 0.022 | 0.429 | 57.33 | 0.536 |
| *Lemniscomys zebra* | 0.221 | 0.093 | 0.457 | 46.13 | 0.697 |
| *Leopoldamys edwardsi* | 0.223 | 0.119 | 0.468 | 44.58 | 0.695 |
| *Mastomys erythroleucus* | 0.247 | 0.150 | 0.493 | 42.78 | 0.711 |
| *Mastomys kollmannspergeri* | 0.257 | 0.164 | 0.503 | 46.94 | 0.720 |
| *Mastomys natalensisv* | 0.250 | 0.154 | 0.495 | 48.45 | 0.713 |
| *Maxomys surifer* | 0.233 | 0.148 | 0.488 | 42.37 | 0.696 |
| *Mus caroli* | 0.241 | 0.113 | 0.470 | 44.41 | 0.710 |
| *Mus cervicolor* | 0.253 | 0.158 | 0.495 | 47.40 | 0.704 |
| *Mus cookii* | 0.241 | 0.120 | 0.473 | 39.59 | 0.712 |
| *Mus mattheyi* | 0.237 | 0.115 | 0.467 | 48.42 | 0.706 |
| *Mus musculus* | 0.232 | 0.044 | 0.446 | 54.72 | 0.539 |
| *Niviventer fulvescens* | 0.228 | 0.108 | 0.463 | 51.14 | 0.686 |
| *Praomys sp. DB-2012* | 0.257 | 0.180 | 0.511 | 43.35 | 0.696 |
| *Rattus exulans* | 0.235 | 0.127 | 0.477 | 42.28 | 0.694 |
| *Rattus losea* | 0.225 | 0.108 | 0.465 | 42.01 | 0.697 |
| *Rattus norvegicus* | 0.171 | -0.028 | 0.402 | 54.07 | 0.420 |
| *Rattus tanezumi* | 0.223 | 0.091 | 0.456 | 47.46 | 0.659 |
| *Thryonomys swinderianus* | 0.256 | 0.183 | 0.520 | 47.42 | 0.735 |
| *Peromyscus eremicus* | 0.183 | -0.003 | 0.430 | 51.31 | 0.413 |
| *Microtus pennsylvanicus* | 0.234 | 0.102 | 0.472 | 53.55 | 0.561 |
| *Peromyscus californicus* | 0.231 | 0.021 | 0.433 | 54.44 | 0.445 |
| *Arvicola amphibius* | 0.236 | 0.054 | 0.453 | 54.44 | 0.515 |
| *Microtus oregoni* | 0.242 | 0.052 | 0.451 | 53.15 | 0.572 |
| *Microtus ochrogaster* | 0.242 | 0.057 | 0.454 | 55.01 | 0.524 |
| *Equus asinus* | 0.234 | 0.021 | 0.428 | 53.28 | 0.374 |
| *Equus przewalskii* | 0.241 | 0.072 | 0.460 | 45.11 | 0.717 |
| *Equus caballus* | 0.238 | 0.067 | 0.457 | 52.43 | 0.599 |
| *Equus quagga* | 0.172 | -0.055 | 0.402 | 49.59 | 0.359 |
